# 1296. Customized Learning About Bettering Sterile Insertion (CLABSI): A Look at Reducing Hospital Acquired Line Infections

**DOI:** 10.1093/ofid/ofac492.1127

**Published:** 2022-12-15

**Authors:** Lana Abusalem, Jake Smith, Tyler Fugere, Zachary Tilley, Tatsiana Savenka, Caitlyn L Wright, Hazel K Liverett

**Affiliations:** University of Arkansas for Medical Sciences, Little Rock, Arkansas; University of Arkansas for Medical Sciences, Little Rock, Arkansas; UAMS, Little Rock, Arkansas; University of Arkansas for Medical Sciences, Little Rock, Arkansas; University of Arkansas for the Medical Sciences, Little Rock, Arkansas; University of Arkansas for Medical Sciences, Little Rock, Arkansas; University of Arkansas for Medical Sciences, Little Rock, Arkansas

## Abstract

**Background:**

Central line associated bloodstream infections (CLABSIs) are associated with significant clinical and economic impacts. A large portion of central venous lines (CVLs) placed at the University of Arkansas for Medical Sciences are done so by resident physicians. A formal education session focusing specifically on aseptic technique for residents has not been utilized so far.

**Methods:**

Literature review and expert opinion was used to guide development of a multi-modal resident education session. The session consisted of a PowerPoint presentation, a video tutorial, and a CVL insertion checklist. More than 50% of the time was allotted to a hands-on session led by residents demonstrating how to aseptically place a CVL under the supervision of an Infection Preventionist. A Likert scale was utilized to assess the level of confidence residents had regarding eight CLABSI prevention metrics both before and after attending the session. Additionally, residents were polled to assess their preferred teaching modality. 66 internal medicine residents and 18 internal medicine-pediatrics residents participated in the study over a five-week period. The results were assessed for statistical significance with a two-tailed Mann-Whitney U-test.

**Results:**

Responses were received from 54 residents prior to the simulation lab session, and from 56 residents after the simulation lab session. The total resident response rate was 66.67%. A statistically significant improvement in resident confidence was found in all eight surveyed CLABSI prevention metrics. The majority of responding residents found workshop style sessions, peer-to-peer teaching and educational videos to be the most useful modalities to deliver information.

Bar Graph Demonstrating a Side by Side Comparison of Resident Reponses Pre- and Post-Intervention

**Figure:**
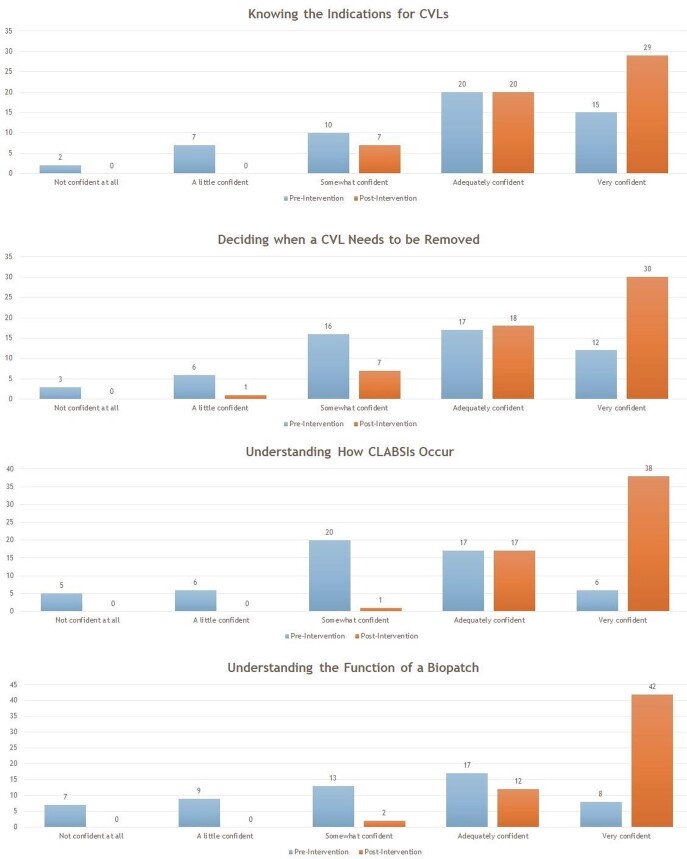

Bar Graph Demonstrating a Side by Side Comparison of Resident Reponses Pre- and Post-Intervention

**Figure:**
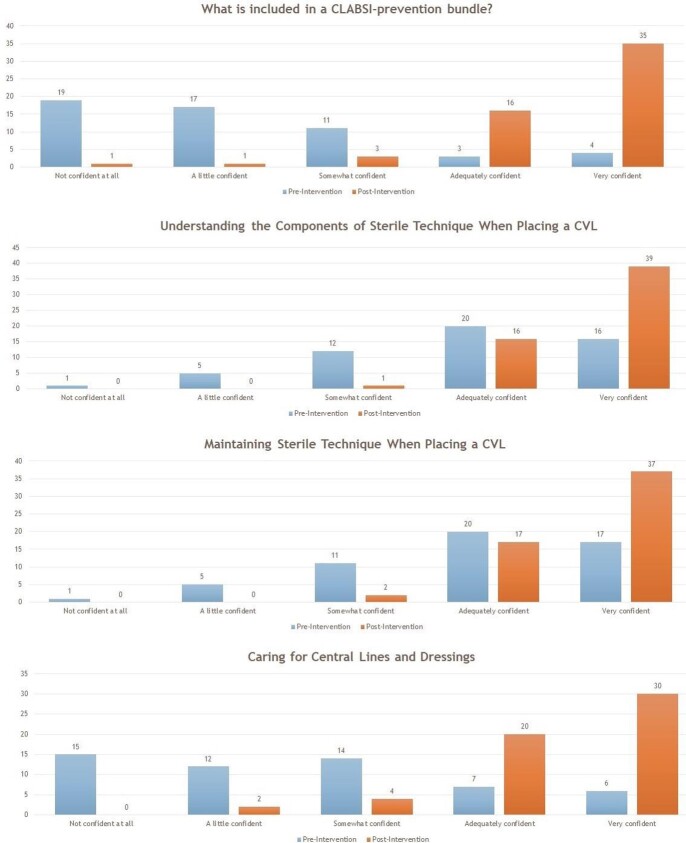

Resident Responses to Preferred Methods of Learning about CLABSI's Pre- and Post-Intervention

**Figure:**
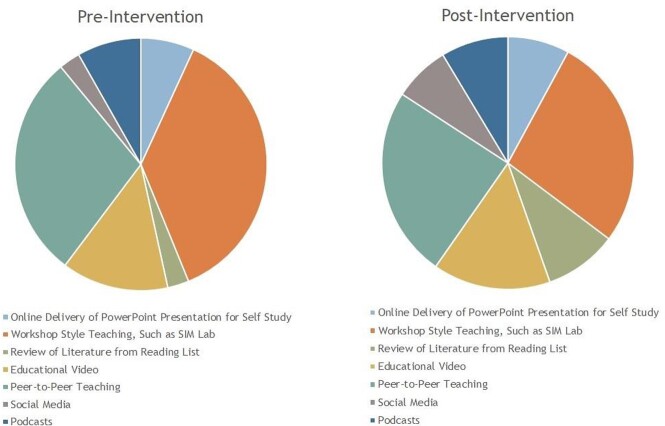

**Conclusion:**

Attendance of a two-hour multimodal resident-driven educational session improved the confidence of residents in their knowledge and implementation of CLABSI prevention metrics. Participants preferred learning via workshop style sessions and educational videos. We recommend a multi-modal approach to resident education, and the consistent involvement of an Infection Preventionist. We hope to expand our curriculum to involve residents in other departments and explore the variation in preferred learning modalities and its overall impact on hospital CLABSI rates

**Disclosures:**

**Hazel K. Liverett, MD**, Merck: Stocks/Bonds.

